# Time to Develop and Predictors for Incidence of Tuberculosis among Children Receiving Antiretroviral Therapy

**DOI:** 10.1155/2021/6686019

**Published:** 2021-11-13

**Authors:** Fassikaw Kebede, Tsehay Kebede, Birhanu Kebede, Abebe Abate, Dube Jara, Belete Negese, Tamrat Shaweno

**Affiliations:** ^1^Woldia University, College of Health Science, School of Public Health, Department of Epidemiology & Biostatics, Ethiopia 2021; ^2^Bahir Dare University, Faculty of Social Science, Department of Geography & Environmental Studies, Ethiopia 2021; ^3^Pawe Woreda Agriculural Inpute and Production Team Leaders, Metekel Zone, Pawe Woreda, North West, Ethiopia; ^4^Debre Markos University, College of Medicine and Health Sciences, Department of Public Health 2021, Debre Markose, Ethiopia; ^5^Debre Birhan University, College of Medicine and Health Sciences, Department of Nursing 2021, Debre Birhan, Ethiopia; ^6^Jimma University Institute of Health Science, Faculty of Public Health, Department of Epidemiology 2021, Jimma, Ethiopia

## Abstract

Infection by the human immune deficiency virus (HIV) is the strongest risk factor for latent or new infection of tuberculosis (TB) through reduction of CD4 T-lymphocytes and cellular immune function. Almost one-third of deaths among people living with HIV are attributed to tuberculosis. Despite this evidence, in Ethiopia, there is a scarcity of information regarding the incidence of tuberculosis for children living with HIV. Thus, this study assessed time to develop and predictors for incidence of tuberculosis in children attending HIV/AIDS care in public hospitals: North West Ethiopia 2021. *Methods*. A facility-based retrospective cohort study was conducted among 421 seropositive children on antiretroviral therapy in two hospitals between January 1, 2011 and December 31, 2020. EPI-DATA version 3.2 and STATA/14 software were used for data entry and analysis, respectively. Tuberculosis-free survival time was estimated using the Kaplan-Meier survival curve. Bivariate and multivariable Cox regression model was fitted to identify predictors at a *P* value <0.05 within 95% CI. *Results*. In the final analysis, a total of 421 seropositive children were included, of whom, 64 (15.2%) developed tuberculosis at the time of follow-up. The mean (±SD) age of the children was 10.62 ± 3.32 years, with a median (IQR) time to develop TB that was 23.5 (IQR = ±19) months. This study found that the incidence of tuberculosis was 5.9 (95% CI: 4.7; 7.6) per 100 person-years (PY) risk of observation. Cases at baseline not taking cotrimoxazol preventive therapy (CPT) (AHR = 2.5; 95% CI, 1.4-4.7, *P* < 0.021), being severely stunted (AHR = 2.9: 95% CI, 1.2-7.8, *P* < 0.03), and having low hemoglobin level (AHR = 4.0; 95% CI, 2.1-8.1, *P* < 0.001) were found to be predictors of tuberculosis. *Conclusion*. A higher rate of tuberculosis incidence was reported in our study as compared with previous studies in Ethiopia. Cases at baseline not taking cotrimoxazol preventive therapy (CPT), being severely stunted, and having low hemoglobin (≤10 mg/dl) levels were found to be at higher risk to developed TB incidence.

## 1. Introduction

The human immune deficiency virus (HIV) is the strongest risk factor for latent or new infection of tuberculosis (TB) through reduction of CD4 T-lymphocytes and cellular immune function [1].

The coinfections of TB/HIV are bidirectional, and the diseases reinforce each other, in that HIV sustains the progression of latent tuberculosis bacilli into active TB, while tuberculosis accelerates the progression of HIV disease to its advanced clinical stage [1, 2]. In 2019, there were an estimated 10.1 million new cases of TB and 1.7 million new deaths, making TB the leading cause of death from a single infectious agent (ranking above HIV/AIDS) [3]. In fact TB is responsible for one–third of TB/HIV-associated deaths for children living with HIV [4, 5]. The burden of this disease is higher in sub-Saharan Africa, where HIV remains a significant problem with inadequate coverage with antiretroviral drugs [6]. Globally, about 36.7 million patients were living with HIV/AIDS, and 2.1 million people became newly infected in 2015. The sub-Saharan Africa countries account for the largest proportion, with 25.6 million people living with HIV [1, 3]. Likewise, in 2018, there were about 251,000 deaths from TB among PLWHIV, which accounts for 33% of total deaths associated with HIV, which is much higher than the case fatality rate expected by WHO, which is ≤5% [1, 7]. Despite different interventions, TB incidence rate in children on ART is high in different settings at different times. Notably, 0.28 per 100 person-years reported in Latin America [8], 1.9 per 100 person-years reported in Zambia [9], 4.0 and 21.1 per 100 person years in South Africa [10], and 17.4 in East Africa [11]. Often in resource-limited settings, estimates of TB in children have been based on extrapolation from adult data [4], but children with TB differ significantly from adult TB patients in their immunological and pathophysiological responses [7].

The Ethiopian Federal HIV and AIDS Prevention and Control Office estimated that the single national HIV/AIDS is among the top ten high burden counties with an incidence rate of 341/100,000 of which 31% of TB patients are living with HIV [5, 7]. Still now, TB/HIV coinfection is the leading cause of death in people living with HIV/AIDS [12]. Although the incidence of TB among adult HIV patients has been exhaustively studied, it is overlooked for HIV-positive children receiving antiretroviral therapy. So, this study was intended to determine the incidence rate and predictors for the time to developed TB among HIV-positive children receiving antiretroviral therapy in North West, Ethiopia 2021.

## 2. Methods

### 2.1. Study Design, Area, and Populations

**Figure 1:**
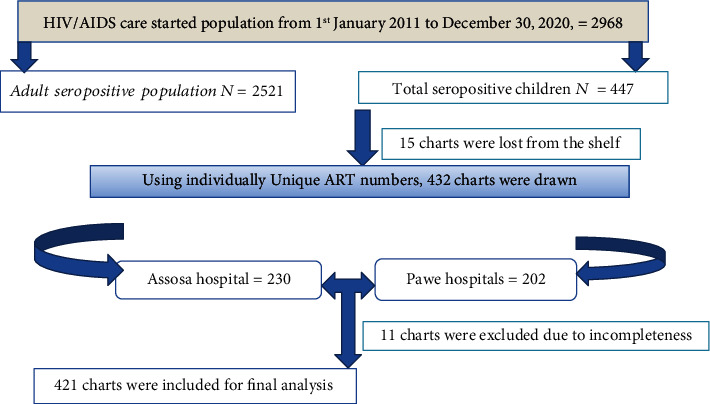
Schematic presentation of sampling procedure from the two general hospitals.

Facility-based retrospective cohort study was employed among 421 seropositive children from 1^st^ January 2011 to December 31/2020 in two (Assosa and Pawe) general hospitals. Both are located in the Benishangul Gumuz region, Northwest of Ethiopia [13]. Apart from other services, these hospitals have been providing ART follow-up care services since 2007. In the hospital, the recorded number of HIV-positive people starting ART care was 2968, of who were 447 HIV-positive children files were there since January 1, 2011 and December 31, 2020 (Figure 1).

### 2.2. Sample Size Determination and Sampling Techniques

The sample size was calculated by using the formula for survival analysis by [14] considering two-sided significance level (*α* = 5%), *Z*_*a*_/2 = *Z* value at 95%confidence interval = 1.96, power (*Z*_*B*_) = 80%, and *P* = %cumulative occurrence of TB incidence, 1.65 HR [15].

(1) $$\begin{array}{c} \begin{array}{c} \text{The final sample size}\;\left(n\right)=\frac{\text{Event}}{P\left(\text{Event}\right)}=\frac{\left(Z_{a/2}+Z_{B}\right)2}{\theta^{2}p\left(1-p\right)}=\frac{\left(\;za/2+ZB\right)}{p\left(1-p\right)\left(\text{lnHR}\right)2},\\ \theta =\ln\left(\text{HR}\right),\\ \text{HR}=e^{\theta }, \end{array} \end{array}$$

where alpha is (*a*) = 0.05, beta is (*β*) = 0.2, AHR is the hazard ratio, *E* is the number of event, and *N* is the (sample size) = *E*/*P* (*E*), where *P* (*E*) = probability of event, and *P* is the cumulative occurrence of treatment failure using values from reference for sample size calculation from two [16]. The final sample size was determined as 430 after adding 10% contingency for incompleteness. From 1^st^ January 2011 to December 31/2020 in the two general hospitals, 447 children started ART care. We included all without any sampling procedure.

Exclusion criteria: patients taking anti-TB treatment at the time of HIV/AIDS enrolment were excluded from the study.

### 2.3. Outcome Ascertainment

The new incidence of TB considered as an event of interest, which is defined as the occurrence of TB in HIV-positive children during successive follow-up at any time after enrollment in the pediatrics HIV care clinic. Children who were lost, died, transferred out, or did not develop the events until the last visit were considered censored, whereas variables including sociodemographic such as age, sex, residence, family size, parental history of TB contact, plus clinical factors like WHO clinical stage, baseline cluster of differentiation (CD4 count), Hgb, functional status, and nutritional status like (stunting, wasting, underweight, MUAC) were considered as independent variables.

### 2.4. Operational Words

Smear-positive pulmonary tuberculosis: at least one sputum smear examination is positive for acid fast bacilli (AFB) by direct microscopy. Smear-negative pulmonary tuberculosis: sputum specimens negative for AFB and radiographical abnormalities were consistent with active TB and the decision by a clinician to treat with a full course of antituberculosis chemotherapy. Extra Pulmonary TB: defined as tuberculosis outside the lung usually results from hematogenous dissemination. Sometimes infection directly extends from an adjacent organ. Symptoms vary by site but generally include fever, malaise, and weight loss. Diagnosis is most often by sputum smear and culture and, increasingly, by rapid molecular-based diagnostic test. Serpositive children: children who had human immune deficiency virus (HIV) in their blood and catgories under age less than 15 years.

### 2.5. Data Collection Instruments and Quality Assurance

A structured English version checklist was developed and used for data extraction from the patients' medical records sheet and Federal Ministry of Health Pediatrics antiretroviral therapy (ART) follow-up form [17]. Four diploma nurses and two degree nurses were recruited for data collection and supervision. One day of training was given for data collection and supervision procedures. A pretest was conducted on 5% of the final sample size at Felege Selam health center to check the reliability of the checklist. After the pretest, necessary modification of the data collection tool was made. Strict follow-up and supervision were carried out during data collection by the principal investigators, and feedback was given daily.

### 2.6. Data Processing and Analysis

EPI-DATA version 3.2 and STATA/14 software were used for data entry and analysis, respectively. Proportional hazard assumption was checked for each variable, and no variable was found with Schoenfeld residual test < 0.05. Categorical variables at bivariable Cox regression were assessed for candidates transferred at *P* value <0.25 for final multivariable Cox regression models, and variables associated with TB incidence in 95% CI at *P* < 0.005 were claimed as the predictor.

### 2.7. Ethical Approval and Consent of Participants

Ethical clearance and ethical approval were obtained from the research institute review board (IRB) of Debre Markose University with reference (Refill no: DMU IRB-984/118/13). A formal letter was submitted to all three hospitals for permission to be done. Debra Markose University waived consent from caregivers in addition to the national research, and ethical review guide waived consent for secondary files.

## 3. Results

### 3.1. Baseline Sociodemographic and Clinical Characteristics

After excluding 11 individuals' files due to incompleteness, we reviewed a total of 421 charts registered from 1^st^ January 2011 to December 31, 2020 as depicted (Figure 1). The mean (± SD) age of study participant was 10.6 ± 3.3 years. More than three-fifths of 261 (62%) participant children were aged ≥11 years, and the majority 219 (52%) of them were from the rural area. About 57.5% of participant had both parents (Table 1).

### 3.2. Baseline Clinical, Hematological, and Laboratory Characteristics

Of the total, 128 (30%) and 89 (21%) of participant children were found to be in clinical world health organization stages (WHO) I and III, respectively. Nearly two-thirds, 263 (62%), of the children had hemoglobin ≥ 10 mg/dl, whereas the majority, 317 (75%), of participants had CD4 cell count ≥ 201 cell/mm^3^. Moreover, 316 (75%) of children took cotrioxzol prophylaxis (CPT). Nearly two in five, 168 (39%), participant children missed isoniazid preventive therapy. Nearly one in five, 81 (19%), study participants shifted baseline ART regimens. The frequent reason was due to toxicity and TB incidence (24/81) and (22/81), respectively. Thirty nine percent of the children had an opportunistic infection. Bacterial pneumonia 53 (35.9%) and PCP 27 (21.6%) were the commonest. Of the total, 147 (35%) had hemoglobin ≤ 10 mg/dl, and more than two-thirds 276 (66%) children vaccinations. Majority, 338 (80%), of cases were in the appropriate developmental stage, while 56 (13.4%) children had poor ART adherence (Table 1).

### 3.3. Baseline Nutritional Status of HIV-Infected Children

Of the total, 33 (8%) and 72 (17%) cases had severe stunting and moderate wasting, respectively. However, 303 (72.3%) participants were on normal percentiles of weight for age > −2 *Z* score (Table 2).

### 3.4. Time to Developed Tuberculosis

In this study, a total of 421 study participants were followed for a different period, contributing a cohort of 1043.1 PY of observation with minimum and maximum of observation 4 to 98 months.

During the follow-up period, 64 (15.2%) individuals were devloped new tuberculosis. this makes the overall incidence density rate(IDR) particicpant children was found 5.9 (95% CI: 4.7; 7.7) per 100 person-years of risk observation. Majority, 44 (70%), of new cases occurred after ART that started 3 years later. About 44 (69%) cases were EPTB, and the remaining 19 (30%) were PTB (Table 3).

**Figure 2:**
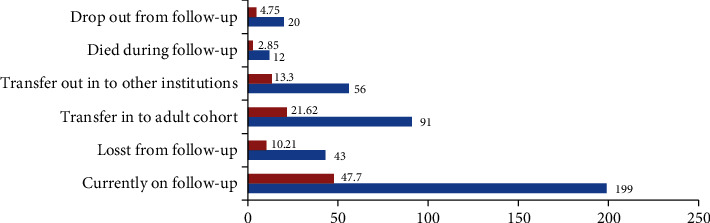
Seropositive children started HIV/AIDS care status in two public hospitals during data collection in public January 2011 to December 31/2020, North West Ethiopia (*N* = 421).

At the end of follow-up, 357 (85%) participant children were censored (excluded), among these 199 (47%) were on follow-up, 91 (21.6%) transferred into adult cohort, and 12 (4.7%) cases died **(**Figure 2).

### 3.5. Kaplan Meier TB Free Probability

**Figure 3:**
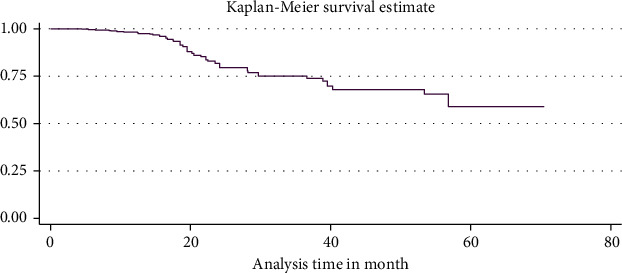
Overall Kaplan Meier survival estimate for all HIV/AIDS care seropositive children care started in two hospitals between 2011 and 2020 North West Ethiopia 2021 (*N* = 421).

**Figure 4:**
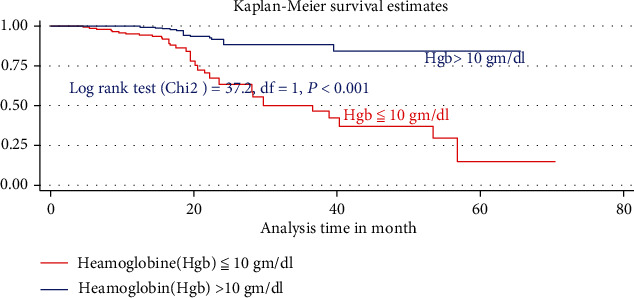
Kaplan Meier estimate of tuberculosis-free survival probability based on levels of hemoglobin for seropositive children, North West Ethiopia 2021.

**Figure 5:**
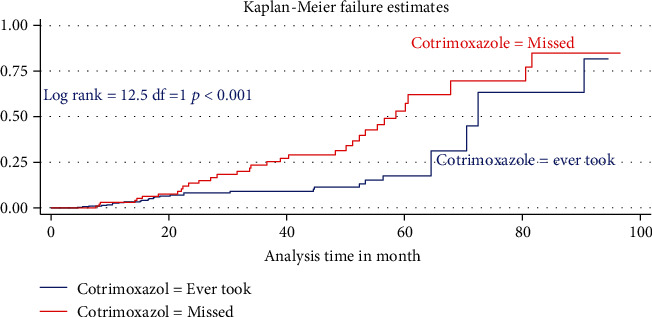
Difference in hazard for tuberculosis incidence in children not taking CPT seropositive children received ART care in two hospitals North West Ethiopia 2021.

The median duration of TB-free probability time was found 35 (IQR = ±29) months. The mean TB-free survival time of the entire follow up was 74.5 months (95% CI: 73.8, 84.3 months). Tuberculosis-free survival probability by the end of the follow-up was determined as 28.5% (95% CI; 22.3-36.9%) (Figures 3–5).

### 3.6. Predictors for Tuberculosis Incidence

In the final multiVariabel Cox regression model, only three variables found significantly associated with time to developed TB. Of this, the risks of developing TB for children who were not taking cotrimoxazole preventive therapy (CPT) were nearly three times higher as compared participant children who took CPT (AHR = 2.5: 95% CI,1.84-4.74, *P* < 0.021). Likewise, the risks of acquiring TB infection for seropositive children being nutritionally curve (height for age (HFA) ≤ −3 *Z* score) or being severely stunted were three times (AHR = 2.9: 95% CI,1.2-7.8, *P* < 0.03) higher than as compared with participant children having normal percentiles (HFA, >−2 *Z* score). Hemoglobin levels had a high predictive value for incident TB; indeed, baseline hemoglobin ≤ 10 mg/dl was four times increase the hazard of developing TB as compared to children having ≥10 mg/dl (AHR = 4.02: 95% CI, 2.1-8.1, *P* < 0.001) (Table 4).

## 4. Discussion

This study is aimed at assessing TB incidence rate and its predictors in children on ART. Accordingly, at the end of the study period, 63 (15%) participants developed new TB incidence, making the overall incidence density rate 5.9 cases per 100 person-years (95% CI: 4.68; 7.68). This is higher than reported from Southern Ethiopia 2.6/100 PYs [18], Debre Markos 2.63/100 PYs [19], Northern Ethiopia 4.2/100 PYs [16], and Gonder 4.9/100 PYOs [20] but lower than findings in Adama hospitals 6.03/100 PYs [21] and South Africa 21.1/100 PYO [10]. This difference might be due to the higher burden of tuberculosis in resource-limited settings [1]. HIV was the immune system and acceleration viral replication responsible depletion of CD4 count [7] and associated with precipitation of new episode for opportunistic infections. This can be reduced early by addressing CPT and IPT, which are inexpensive and highly effective for reducing loads of endogenous reactivation of latent TB [22]. However, being seropositive children who missed CPT were significantly associated with risks of developed TB incidence. This finding is comparable with those reported in Gonder hospital [16] and Adam hospital [20]. The finding of this research also indicated that HIV-infected children having severe stunting were independently associated with the incidence of TB as compared with HIV-infected children who have no stunting. This is in line with the finding in Adama [21], Tanzania [23], Uganda, and Zimbabwe [9]. This might be due to HIV infection increasing nutrient malabsorption due to metabolic alterations that culminate in weight loss and stunting with time leading to early exposure for opportunistic infections [24]. Similarly, the existence of rapid viral replication consumed body energy and creates an arena for the incidence of TB [25, 26]. This finding also showed that children having hemoglobin ≤ 10 mg/dl were independently associated with TB incidence as compared with HIV-infected children having hemoglobin levels > 10 mg/dl. This is in line with the study finding in Adama hospital [21], Gonder hospital [20], Northern Ethiopia [16], Dar es Salaam, Tanzania [23], and England [27]. In fact, this is due to hemoglobin levels having a high predictive value for incident TB and death. TB incidence is directly associated with severe anemia [28]. Regardless of ART, moderate or severe anemia during ART follow-up can be an independent predictor for TB [28, 29].

### 4.1. Limitation of the Study

The retrospective nature of this study is one of the limitations of this study. Due to this, some of the clinically important predictor variables that have been independently associated with the incidence of TB occurrence in other studies, like the educational status of children and economic status of the family, were not included in this study.

## 5. Conclusion

Tuberculosis incidence was high among seropositive children attending HIV/AIDS care, especially in the first three years after ART initiated as compared with that of subsequent years, since more than half of 44 (69%) new cases of TB occurred. Levels of hemoglobin, missed cotrimoxazole preventive therapy, and nutritionallysevere stunting (HFA = ≤−3 *Z* score) were significantly associated with the incidence of tuberculosis. Besides, intensified screening for provisions of isoniazid preventive therapy to children living with HIV/AIDS is highly recommended.

## Figures and Tables

**Table 1:** Baseline sociodemographic, clinical, and laboratory characteristics of children received ART in two public hospitals (between = 2009 and 2018).

Variables	Categories	Numbers (total = 421)	Percent
Sex	Male	204	49
	Female	217	51

Age	≤5 years	46	11
	Between 6 and 10	114	27
	≥11 years	261	62

Residence	Urban	205	48
	Rural	216	51

Family size	≤2	133	31
	3-4	219	52
	5-6	50	11
	≥7	19	5

Hemoglobin	>10 mg/dl	263	63
	≤10 mg/dl	158	38

WHO	Stage I	128	30
	Stage II	147	35
	Stage III	89	21
	Stage IV	57	13

CD4 count	<100	30	7
	101-200	74	7
	≥201	317	75

Functional status	Working	307	72
	Ambulatory	72	17
	Bedridden	42	10

Adherence	Good	224	59
	Fair	121	29
	Poor	56	13

Isoniazid	Took	258	61
	Missed	163	38

Cocotrimoxazole	Took	321	76
	Missed	100	23

Opportunistic infections	Present	126	29
	Absent	295	70

Vaccination	Completed	276	66
	Not updated	76	18
	Defaulted	69	16

TB contact history	Yes	135	32
	No	286	68

Change of AR regiment	Present	85	20
	Absent	336	79

Parental status	Both parent alive	242	57
	Paternal orphan	115	27
	Maternal orphan	38	9
	Both parent orphan	26	6

**Table 2:** Baseline nutritional status of seropositive children attending HIV/AIDS care in two public hospitals North West Ethiopia 2021 (*N* = 421).

Nutritional parameter	Frequency	Percent	chi^2^(2)	*P* < 0.05
Weight for height (WFH) normal (>-2 *Z* score)	304	72	7.5724	0.023
Moderate wasting ≤ −2 − 3 *Z* score	71	17		
Severe wasting ≤ −3 Z score	46	11		
Height for age (HFA) normal (>-2 *Z* score)	284	68	5.9262	0.052
Moderate stunting ≤ −2 − 3 *Z* score	104	5		
Severe stunting ≤ −3 *Z* score	33	8		
Weight for age(WFA) normal (>-2 *Z* score	277	66	9.1780	0.010
Moderate underweight ≤ −2 − 3 *Z* score	115	27		
Severe underweight ≤ −3 *Z* score	29	7		

**Table 3:** Time to developed TB incidence for seropositive children attending HIV/AIDS care in public hospitals between January 2011 and December 31/2020, North West Ethiopia (*N* = 421).

Years	No. of children at started	Withdrawn during year	At risk children	No. of TB case (63)	TB incidence	Cumulative probability	Survival rate	95% CI
>1 year	421 (100)	13 (20.6)	408(96.9%)	13 (20.6)	13 (3.1%)	13 (20.6%)	96.7%	94.5-98.1
2 years	408 (96.9%)	18 (28.5%)	390(92.6%)	18 (28.5%)	18 (4.3%)	31 (49.2%)	90.5%	86.5-93.2
3 years	390 (92.6%)	8 (12.6%)	382(90.7%)	8 (12.6%)	8 (1.99%)	39 (61.8%)	86.3%	81.5-89.9
4 years	382 (90.7%)	5 (7.93%)	377(89.5%)	5 (7.93%)	5 (1.15%)	44 (69.7%)	82.5%	76.5-87.1
5 years	377 (89.5%)	12 (19.0%)	365(86.6%)	12 (19.0%)	12 (2.8%)	56 (88.7%)	61.9%	49.5-72.8
6 years	365 (86.6%)	4 (6.34%)	361(85.7%)	4 (6.34%)	4 (0.98%)	60 (95.1%)	38.9%	29.1-57.5
7 years	361 (85.7%)	2 (3.17%)	359(85.2%)	2 (3.17%)	2 (0.047%)	62 (98.2%)	29.9%	26.5-46.4
End of follow	358 (84.8%)	1 (1.72%)	358(84.8%)	1 (1.72%)	1 (0.023%)	63 (100)	28.5%	22.3-36.9
Total		63 (100)	—	63 (100)	—	100	—	—

**Table 4:** Bi-variable and multivariable Cox-proportional hazard regression for predictors of TB among children treated on HIV/AIDS care in two public hospitals 2021 (*N* = 421).

Variables	Categories	COR 95% CI	AOR=95% CI	P< 0.05
Age of children	**< =5 years**	**1**	**1**	
	**6-10 years**	**3.4 ( 1.09 - 5.5)**	**0.92 (0.28; 2.94)**	**0.82**
	**>=11 years**	**3.6.(1.7 - 5.6)**	**1.2 (0.32; 2.8)**	**0.12**
IPT	**Given**	**1**	**1**	
	**Missed**	**2 .6(2.1 - 8.3)**	**1.3(0.79 - 2.2)**	**0.08**
CPT	**Given**	**1**	**1**	
	**Missed**	**3.7(2.8- 7.8)**	**2.5(1.28; 5.3)**	**0.021**
TB history of contact	**Yes**	**1.2(1.08 - 3.4)**	**1.09 (0.8 - 2.2)**	**0.11**
	**No**	**1**	**1**	
Vaccination	**Complete**	**1**	**1**	
	**Not updated**	**2.1 (0.9 - 2.7)**	**1.2(0.72; 1.5)**	**0.40**
	**Defaulted**	**1.7(1.2- 2.8)**	1.3(0.8; 1.9),	**0.12**
Adherence	**Good**		**1**	
	**Fair**	**2.1(1.7 -- 4.1)**	1.8(0.87; 2.9),	**0.11**
	**Poor**	**3.1(2.05 -6.3)**	**1.2 (0.93- 2.7)**	**0.06**
HFA>-2 Z score	**No stunting**	**1**	**1**	
	**Moderate stunting**	**2.1(1.5 -- 2.8)**	**1.2(0.8; 2.7)**	**0.17**
	**Sever stunting**	**3.9(3.8 --5.1)**	**2.9(1.2; 7.8)**	**0.03**
WHO clinical	**stage I&II**	**1**	**1**	
	**Stage III&IV**	**9.1(4.9 16.4)**	**1.3 (0.73-2.5)**	**0.12**
Hemoglobin	**> 10 mg/dl**	**1**	**1**	
	**≤10 mg/dl**	**9.62(5.13 18.0)**	**4.02(2.1- 8.1)**	**0.001**

## Data Availability

The data of this original research are available from the corresponding author upon reasonable request.

## Abbreviations

HIV: Human immune deficiency syndrome
TB: Tuberculosis
AHR: Adjusted hazard ratio
PLWHIV: People living with HIV
WFH: Weight for height
WFA: Weight for age
HFA: Height for age
PYOs: Person years of observations
LLH: Log likely hood ratio.
